# Whole Mitochondrial Genome Sequencing Analysis of Canine Testicular Tumours

**DOI:** 10.3390/ijms25189944

**Published:** 2024-09-14

**Authors:** Angelika Tkaczyk-Wlizło, Krzysztof Kowal, Anna Śmiech, Brygida Ślaska

**Affiliations:** 1 Institute of Biological Bases of Animal Production, University of Life Sciences in Lublin, Akademicka 13 St., 20-950 Lublin, Poland; angelika.tkaczyk@up.lublin.pl (A.T.-W.); krzysztof.kowal@up.lublin.pl (K.K.); 2 Department of Pathomorphology and Forensic Medicine, Faculty of Veterinary Medicine, University of Life Sciences in Lublin, Głęboka 30 St., 20-612 Lublin, Poland; anna.smiech@up.lublin.pl

**Keywords:** domestic dog, neoplasm, mtDNA, SNP, heteroplasmy, VNTR, testes

## Abstract

Currently, the molecular background based on mitochondrial DNA (mtDNA) analysis of canine testicular tumours is underestimated. The available data mostly focus on histopathological evaluations, with a few reports of nuclear genome (nDNA) studies. Tumourigenesis represents a highly complex and diverse genetic disorder, which can also encompass defects in mtDNA. The aim of this study was to identify molecular changes in whole mitochondrial genome sequences obtained from dogs affected by testicular tumours. Samples of blood, tumour, and healthy tissue were collected from each animal, and mtDNA (ultimately 45 samples) was subsequently sequenced. Thereafter, protein analyses were performed to assess the impact of the identified molecular alterations on the amino acid level. The total number of observed changes included 722 SNPs, 12 mutations, 62 indels, 5 indel mutations, and 35 heteroplasmic sites. The highest number of mtDNA variants in protein-coding genes *COX1*, *COX3*, *ATP6*, *ND1*, *ND4*, and *ND5* was observed. Interestingly, SNPs were found in 10 out of 22 tRNA genes. Most of the identified mtDNA defects were synonymous changes at the amino acid level. Also, polymorphisms and heteroplasmy were frequently observed in the variable number of tandem repeat (VNTR) regions, especially in its fragment spanning 16,138–16,358 bp. Based on the obtained results, it was possible to select 11 polymorphisms that occurred in all the tested samples (benign, malignant) and an additional five SNPs identified only in benign neoplasms. The comprehensive analysis of malignant testicular tumours demonstrated a significant diversity in their molecular profiles, with changes ranging from 17 to 101 per sample.

## 1. Introduction

Testes are the fourth most commonly diagnosed site for canine tumourigenesis after skin, soft tissue, and mammary glands. Canine testicular tumours (TTs) represent about 90% of all neoplasms affecting male genitalia. Among testicular neoplasms, sex cord-stromal tumours, Sertoli cell tumours (SCT), interstitial (Leydig) cell tumours (ICL or LCT), and germ cell tumours in the form of seminomas (SEM) constitute a major part of frequently developed neoplasms [1,2,3]. Other types of TTs, such as embryonal carcinoma, mixed germ cell-sex cord-stromal tumour, and teratoma, are rare [4]. The risk of testicular tumours (TTs) increases with age, typically occurring in seniors (average age is 10 years) or geriatric dogs, and is notably higher in animals with cryptorchidism, which is a well-known predisposing factor [3,5,6]. Depending on the research, the development of testicular neoplasms in cryptorchid testes (mostly unilaterally) occurs in 5.9% [1] to 23.2% [5] of canine cases. Increased risk of primary testicular tumours is observed in such dog breeds as Boxers, Collies, German Shepherds, and Shetland Sheepdogs [2,6,7].

The majority of available papers present only histopathological evaluations of canine TTs [1,5,8] or in combination with biochemical analyses [9,10], but little is known about the molecular background of testicular tumour development. To date, only a few nuclear DNA analyses, including such genes as *TP53* and *SIRT1*, have been performed in the case of canine TTs [11]. On the other hand, tumourigenesis is an energy-dependent condition; hence, its development may be related to abnormalities of mitochondrial DNA (mtDNA) [12]. There are many reports on changes in mtDNA in canine mammary tumours [13,14,15], mast cell tumours [16], or transmissible venereal tumours [17,18]. In the case of TTs, this issue has not yet been thoroughly analysed. To the authors’ best knowledge, only one paper, published by Slaska et al. (2016), has reported one case of canine SCT in a German Shepherd, where the *ND4* gene analysis revealed a lack of mtDNA changes [15].

Molecular research is essential for understanding the pathogenesis of canine testicular tumours. This is the first study of 15 cases of canine testicular tumours wherein detailed mtDNA analyses based on whole mitochondrial genome sequencing were performed. The aim of the research was to analyse the mtDNA genomes of affected dogs in combination with a determination of the histopathological types of TTs to identify molecular changes, such as mtDNA polymorphisms, mutations, indels, and heteroplasmy associated with TTs. Additionally, available individual factors, such as age and cryptorchidism, were taken into consideration.

## 2. Results

### 2.1. mtDNA Changes in TTs

The analyses conducted on 45 mtDNA genomes revealed a total of 836 nucleotide changes in mitochondrial genes, including 722 SNPs, 12 mutations, 62 indels, 5 indel mutations, and 35 heteroplasmic sites (Tables S1–S3). Polymorphisms were identified in each of the 13 genes encoding respiratory chain and oxidative phosphorylation. The highest number of SNPs was observed in oxidative phosphorylation (OXPHOS) complexes: IV-cytochrome oxidase: *COX1* gene (80), *COX3* gene (45), V-ATP synthase: *ATP6* gene (55), and I-NADH dehydrogenase: *ND1* (48), *ND4* (64), and *ND5* (57) genes. On the other hand, the lowest number of changes occurred in the *ATP8* (2) gene (Table S3). The vast majority of changes observed in protein-coding genes were found in malignant tumours (69.5%), compared to benign neoplasms (30.5%; Table S4).

Also, 13 nucleotide alterations were observed in 10 out of the 22 tRNA genes. Interestingly, in each sample, indel m.2678_2679insG and polymorphism m.2683G>A were noted in the *tRNA ^Leu^* ^(*UUR)*^ gene (Table 1). The list of mtDNA changes observed in the tRNA genes is presented in Table S6. All changes observed in mtDNA tRNAs were analysed using the Canis MitoSNP database [19]. Variants of *tRNA ^Leu^* ^(*UUR*)^ and *tRNA ^Leu^* ^(*CUN*)^ were found in the DHU arm, whereas the SNP in *tRNA ^Phe^* was located in the DHU loop. Next, four of the 13 changes in the tRNA genes were observed in the TΨC loop region from tRNAs: *tRNA ^Trp^*, *tRNA ^Asn^*, *tRNA ^Arg^*, and *tRNA ^Thr^*. Additionally, a polymorphism in the TΨC arm of the *tRNA ^Asp^* was found. On the other hand, changes in the central loop were observed in *tRNA ^Ser^* ^(*AGY*)^ and *tRNA ^Pro^*, while the only change in *tRNA ^Asp^*, m.6967A>G, was placed in the acceptor arm (Table S6). Twelve SNPs were observed in the *12s rRNA* gene, and 27 SNPs were detected in the *16s rRNA* gene (Table S3). Moreover, one heteroplasmy m.634C/T* in *12s rRNA* and one indel mutation m.1486delAA in *16s rRNA* were identified (Table S5).

Also, the abnormalities of the mtDNA were observed in the non-coding region of the D-loop. The total number of mtDNA changes in the D-loop was as follows: 184 SNPs, 10 mutations, 12 indels, and 4 indel mutations. The majority of SNP polymorphisms were found in HVI (hypervariable part I): 104 (56.5%), followed by VNTR (variable number of tandem repeats: 61 (33.2%) and HVII (hypervariable part II): 19 (10.3%) (Tables S2 and S3). The VNTR region is located between positions 16,130 and 16,430 bp in the D-loop. In this region, 31 heteroplasmic sites were observed, especially between 16,138 and 16,358 bp (25/31; 80.6%). Overall, 86.1% of all heteroplasmic sites and 23.1% of polymorphisms were observed in the non-coding D-loop region. Moreover, 4 out of 15 dogs’ loss of a polymorphic 10-bp repeat unit (5′-GTACACGT(A/G)C-3′) in VNTR was noted (Table S2).

The analysis of the observed mtDNA changes indicated 11 common polymorphisms across all the samples (Table 1). Among them, 7 out of 11 occurred in the protein-coding genes of OXPHOS (*COX1*, *COX3*, *ATP6*, *ND4L*, *ND5*), 2 in *tRNA-Leu* ^(*UUR*)^ (m.2678_2679insG, m.2683G>A), and 2 in the HVI region (m.15639T>G, m.15814C>T) of the D-loop. To analyse the impact on the protein level caused by the observed variants, the SIFT method was used and revealed that the variants noted in the *COX1* (3) and *ATP6* (1) genes resulted in synonymous changes in proteins p.Leu7=, p.Ala32=, p.Gly239=, and p.Leu135=, respectively. These molecular alterations were not related to deleterious effects on the protein level. The following variants identified in the *COX3* gene, p.Cys55Tyr, the *ND4L* gene, p.Met1Val, and the *ND5* gene, p.Ser508Thr, caused non-synonymous changes in the amino acid level, but only the amino acid change in the *ND4L* gene was intolerant (Table 1).

Apart from the commonly observed 11 SNP polymorphisms, protein analyses were conducted for all non-synonymous changes identified in protein-coding genes using the SIFT and SOPMA tools (Table S7). The majority of the analysed changes (78.9%) were tolerant. Among the 12 mutations, only one, m.4303A>G, occurred in the *ND2* gene of the blood sample collected from dog T195. This was a synonymous mutation p.Leu159=, without a deleterious effect on the protein level. The following mutations were found in VNTR in the testicular tumours: m.16158A>G (T104, T137), m.16178A>G (T195), m.16188G>A (T137), m.16198G>A (T87), m.16338G>A (T138), and m.16358A>G (T104). Additionally, mutations in individual samples of blood: m.16348G>A m.16388A>G (T066) and healthy tissue: m.16338G>A (T137) and m.16388A>G (T139) were noted in this region between 16,138 and 16,388 bp.

### 2.2. mtDNA Alterations in Benign TTs

**Figure 1:**
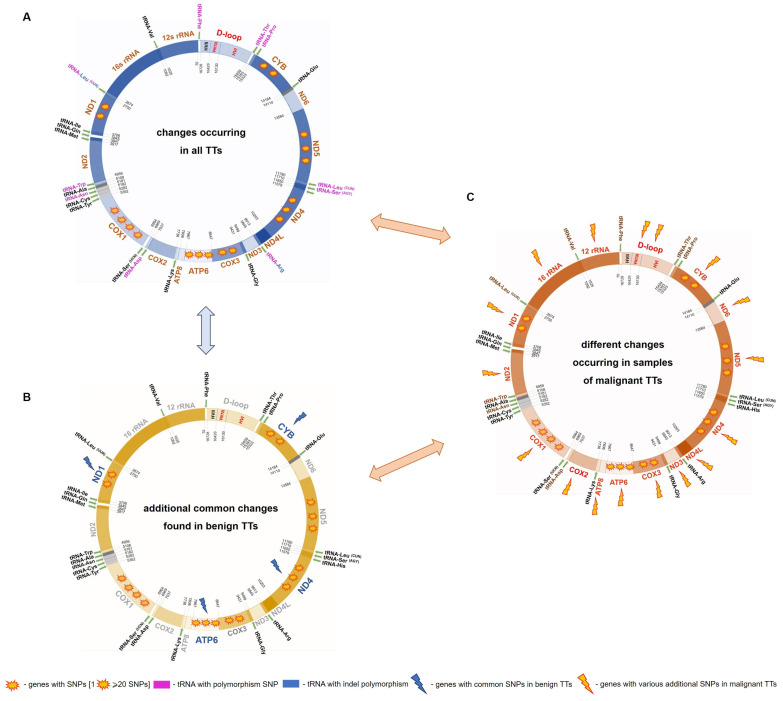
Localisation of changes identified in the mitochondrial genome, including common mtDNA variants occurring in each sample (**A**), additional changes found in each benign TTs (**B**), and different variants found in malignant TTs (**C**).

In this research, 5 out of the 15 dogs had benign testicular tumours, i.e., SCT or LCT (Table 2). The detailed analysis of the obtained results revealed that, in addition to the common changes occurring in each sample, dogs with benign neoplasms exhibited further common mtDNA alterations (not related to the type of TT). The following variants were found in the protein-coding genes: *ND1*: m.2962C>T, m.3196T>C, *ATP6*: m.8281T>C, and *ND4*: m.10992G>A and in *CYB*: m.15214G>A. Interestingly, all of these changes were related to the amino acid codons for leucine or its isomer isoleucine and resulted in synonymous changes (Table 1, Figure 1).

### 2.3. mtDNA Variants of Benign vs. Malignant TTs

The common additional mtDNA alterations in the benign tumours were also observed in a few samples of malignant neoplasms but not in each case. The results obtained from dogs with seminomas confirmed the complexity and variability of malignant tumours (Figure 1). Except for the changes occurring commonly in each mtDNA of affected dogs (11 SNPs, Table S1), no additional common variants were found in the samples of these neoplasms. However, many different individual changes in mtDNA were noted (Figure 1, Table S1). The analyses showed the occurrence of indels (length polymorphisms) in the D-loop: m.15938delG (T139, T15) and m.16664insCC (T163), as well as two indel mutations: m.15938delG (T137) and m.16388delA (T121). However, it should be noted that m.16663insCC was also found in benign TT: T195.

**Figure 2:**
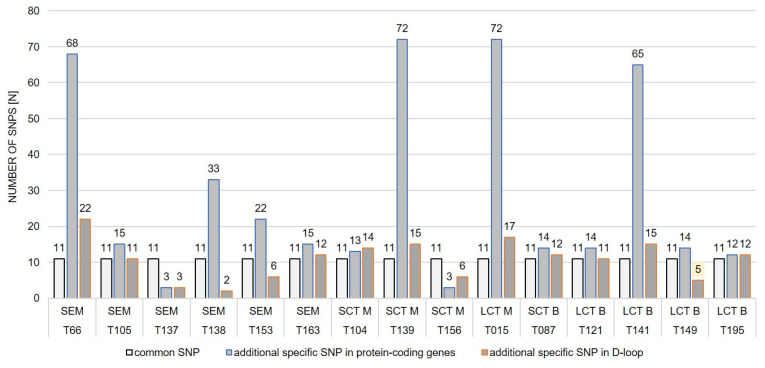
Comparison of the total number of SNPs, including common and additional mtDNA variants considering different types of TTs: SEM—seminoma, SCT M—Sertoli Cell Tumour (M—Malignant), SCT B—Sertoli Cell Tumour (B—Benign), LCT M—Leydig Cell Tumour (Malignant), LCT B—Leydig Cell Tumour (Benign).

The number of additional SNPs in the mtDNA genes, excluding the 11 SNPs common to all the samples, varied between 3 and 72 in the malignant tumours and from 14 to 65 in the benign neoplasms. In the case of the D-loop region, the range was from 2 to 22 in the malignant neoplasms and from 5 to 15 in the benign neoplasms, respectively. The number of SNPs per sample of the malignant TTs was strongly diversified in comparison to the benign neoplasms, for which the number of SNPs in the mtDNA genes was, on average, 14 and 11 in the D-loop region, with one exception of dog T141 (Figure 2).

The difference between malignant and benign samples was also observed in the VNTR region, where 28 and 27 heteroplasmic sites, as well as 18 and 10 polymorphic localisations, were observed, respectively. Next to the D-loop, the heteroplasmy in the malignant tumours was observed in the *12s rRNA* gene: m.634C/T* (T139) and the *ND4* gene: m.11003G/A* (T66). Also, a heteroplasmy (*COX3*, m.9041A/G*) was found in and blood and healthy tissue of dog T105 with malignant LCT.

### 2.4. mtDNA Changes and Characteristics of Dogs

Despite the small group of the tested samples and the limited individual data, an attempt to associate numerous mtDNA changes with the available individual data was made. The comparison between the molecular results and the age groups, as well as the individual ages of affected dogs, did not indicate any association. The high diversity observed in the molecular changes per sample did not increase in the older animals (senior vs. geriatric dogs). Among the different dog breeds, the most numerous were German Shepherds (4/15). These animals were diagnosed with malignant STCs (2) and LCTs (2) (Table 2). However, German Shepherds constituted a small group; hence, no breed-specific mtDNA variants were found. Also, the analysis of cryptorchidism as a well-known risk factor with the molecular results did not indicate any additional mtDNA changes. The comparison between the histopathological type of TTs and the mtDNA variants obtained did not indicate any common polymorphisms. However, the analysis of the neoplasms divided into two groups, benign vs. malignant TTs, showed a few interesting findings.

## 3. Discussion

Testicular tumours are the most common neoplasms of canine male genitalia [2]. Despite the incidence of TTs, only a few predisposing factors, such as cryptorchidism, age, and breed, were determined [2,20]. Currently, the vast majority of analyses of canine TTs were based on histopathological, immunohistochemical [4,20,21], or/and biochemical evaluations [10] without molecular research, which is essential for understanding the pathogenesis.

### 3.1. Instability of Mitochondrial DNA

Mitochondria are crucial organelles which provide energy to cells in the form of ATP through the electron transport chain (ECT) and OXPHOS. However, mitochondria are multitasking organelles participating in, e.g., the metabolism of amino acids, fatty acids, and steroids, the maintenance of Ca^2+^ homeostasis, or the regulation of the cellular redox status [12]. Given their crucial function in steroidogenesis (conversion of cholesterol into pregnenolone), the importance of mitochondria in spermatogenesis is undeniable. Some abnormalities of mitochondria and mtDNA in human testicular tumours and semen quality have been described to date [22,23]. In the case of dogs, there are reports on changes in mtDNA in different canine tumours [14,17,24]. However, with the exception of a single case reported by Slaska et al. [15], no detailed mtDNA analyses of canine testicular tumours have been conducted.

The instability of the mitochondrial genome causes disruptions in energy metabolism via increased glucose metabolism, calcium dysregulation, or altered production of reactive oxygen species (ROS). Consequently, molecular changes in mtDNA are a significant concern in cancer-associated dysfunctions. Furthermore, mtDNA variations contribute to the vast diversity and plasticity of tumours [25,26].

### 3.2. Identified mtDNA Changes

The present analyses showed a tremendous diversity in the mitochondrial genomes of canine TTs, including 722 SNPs, 12 mutations, 62 indels, 5 indel mutations, and 35 heteroplasmic sites (Table S3). The number of SNPs ranged from 3 to 72 in the protein-coding genes and from 2 to 22 in the non-coding region. None of the tested samples had an equal number of changes in the mtDNA (Figure 2). These data indicated high molecular diversification of testicular tumour cells. However, it should be emphasised that a set of commonly occurring polymorphisms: m.2678_2679insG, m.2683G>A, m.5367C>T, m.5444T>C, m.6065A>G, m.8368C>T, 8807G>A, m.9911_9912insGT, m.13299T>A, and m.15639T>G, m.15814C>T was detected in samples from each analysed dog (Table 1). Interestingly, in all the benign TTs, additional five SNPs were indicated: m.2962C>T, m.3196T>C, m.8281T>C, m.10992G>A, and m.15214G>A (Table 1, Figure 1). The detailed analysis showed the occurrence of the five above-mentioned mtDNA changes also in the malignant tumours, but not in each sample. These somatic mtDNA variants, probably arising within the individuals, may belong to the second class of mtDNA variants-mtDNA inducers, which trigger genetic transformation from benign to malignant neoplasms [25].

Apart from the 11 common SNPs occurring in all the samples, the number of additional molecular changes in the protein-coding genes varied from 3 to 72 in the malignant tumours and from 14 to 65 in the benign neoplasms, whereas the changes in the non-coding D-loop region ranged from 2 to 22 in the malignant neoplasms and from 5 to 15 in the benign neoplasms, respectively. Of note, the number of SNPs in the malignant testicular tumours was strongly diversified in comparison to the benign neoplasms, for which the number of SNPs in the mtDNA genes was, on average, 14 and 11 in the D-loop region, with one exception of dog T141 (Figure 2). The increased number of mtDNA changes observed in dog T141 with benign LCT may be explained by the enormous instability of the mitochondrial genome. This dog was in critical condition and, shortly after testicular tumour surgery, underwent euthanasia due to liver failure.

### 3.3. Oxidative Phosphorylation Complex I

The highest number of SNPs was observed in the genes *COX1*, *ND4*, *ND5*, and *ATP6* encoding the OXPHOS complexes. In some cancers, mutual participation of mtDNA and nDNA changes is observed [25]. It is difficult to determine this phenomenon in canine testicular tumours due to the limited research. NADH dehydrogenase complex I is encoded by 41 subunits (34 nuclear and 7 mitochondrial). Dias et al. (2020) reported underexpression of mitochondrial subunits NDUFS1 (nDNA) in human non-seminoma testicular cancers [27]. Moreover, this change in complex I impair sperm quality by dysregulation of an important signalling pathway of spermatogenesis [22]. On the other hand, in the present study, many SNPs were observed in subunits encoded by mitochondrial genes *ND1*, *ND2*, *ND4*, *ND5*, and *ND6* (Table 1, Table S3). Changes in these mtDNA genes were also identified in human [28,29] and canine [30] poor sperm quality research. As described above, male infertility is simultaneously noted with TTs as a result of disturbed steroidogenesis [22].

### 3.4. Comparison of mtDNA Changes of Benign vs. Malignant TTs

It should be emphasised that the vast majority of the identified SNP polymorphisms (502 out of 722; 69.5%) were noted in the malignant testicular tumours in comparison to the number of changes in the benign neoplasms (220/722; 30.5%). The detailed analysis showed that, in the case of each affected gene, the percentage in the group of malicious neoplasms was at least 50% (Table S4). These results highlight the differences in the number and percentage of mitochondrial genes and the non-coding region affected in malignant vs. benign TTs.

It is worth emphasising that among 11 commonly occurring SNPs, 7 were in protein-coding genes and resulted in 3 non-synonymous (p.Cys55Tyr, p.Met1Val, p.Ser508Thr) and 4 synonymous (p.Leu7=, p.Ala32=, p.Gly239=, p.Leu135=) changes. Interestingly, all additional SNPs identified in benign TTs were synonymous and were related to the amino acid codons for leucine (Leu) or its isomer isoleucine (Ile). The observation that changes of Leu or Ile may be frequent in synonymous changes, was also noted by other authors. Bin et al. [31] analysed mutational signatures of synonymous changes across 15 types of human cancers. The obtained results showed that the largest number of synonymous mutations involved codon for Leu.

The analyses of the non-coding D-loop region showed that SNPs more frequently occurred in HVI than in HVII, but the VNTR region was definitely the most heteroplasmic part. The similarity in the localisations of mtDNA changes was also observed in malignant mammary gland (MGT) tumours [13]. However, this is not a common issue. For example, Ziółkowska et al. (2023) analysed solid MGTs and showed that the vast majority of identified SNPs were located in VNTR [24]. In other cases, it is infeasible to compare because the authors described only changes in the HVI region of the D-loop [14,16].

### 3.5. tRNA Variants

Noteworthy, tRNA genes are considered “hotspots’’ for mitochondrial mutations because two-thirds of disease-causing mtDNA mutations occur in tRNAs [32]. This is prominent, especially in canine monogenetic mitochondrial disorders resulting from mtDNA variants, e.g., *tRNA^Tyr^* [32] or *tRNA^Leu^* [33]. The molecular heterogeneity of tumours results in changes in different genes and regions. In this study, common variants of the *tRNA* ^*Leu* (*UUR*)^ gene: m.2678_2679insG and m.2683G>A were found in each tested sample. These polymorphisms were also observed in canine mammary gland tumours [34]. Moreover, additional mtDNA changes were noted in 10 out of 22 tRNA genes, albeit they were polymorphisms, not mutations. The total number of tRNA variants per sample ranged from 2 to 6. The observed variants were related to the changes in the tRNA regions, especially *tRNA ^Phe^*, in *tRNA ^Leu^* ^(*UUR*)^, *tRNA ^Leu^* ^(*CUN*)^ in the DHU arm/loop and *tRNA ^Trp^*, *tRNA ^Asn^*, *tRNA ^Asp^*, *tRNA ^Arg^*, and *tRNA ^Thr^* in the TΨC arm/loop (Table S5). Structural alterations in tRNAs are related with disturbances in translation and contribute to respiratory chain deficiency but only if mutated mtDNA exceeds a certain threshold [32]. To assess this, additional detailed tRNA research is needed.

## 4. Materials and Methods

### 4.1. Samples

This combined prospective and retrospective study was conducted from 2021 to 2023 and included 15 selected cases of canine testicular tumours collected from 2016 to 2020. Blood, tumour tissue, and healthy tissue were collected from each dog for histopathological evaluations and molecular analyses. The healthy tissue was a small fragment of skin obtained during the surgical excision of TTs. In total, 45 samples were collected and analysed. The biological materials from affected dogs were collected according to the approval from the II Local Ethical Committee for Animal Experiments in Lublin, Poland (Resolution number 79/2014).

### 4.2. Patient Eligibility

The inclusion criteria were the age of the dogs (age groups: senior or geriatric), and the histopathological evaluations confirmed the diagnosis of testicular tumours of affected dogs. Only dogs that were diagnosed with TTs but did not undergo chemotherapy or radiotherapy were included in this study. The exclusion criteria were the lack of preserved samples for genetic analysis, the age of the dogs < 7 years (younger than the senior group), and histopathological diagnosis other than SCT, SEM, or LCT. Additionally, samples without basic data such as age, breed, and occurrence of cryptorchidism were excluded.

### 4.3. Sample Information

The study included 15 dogs of different breeds and crossbreeds diagnosed with testicular tumours. The breeds were grouped according to size based on the American Kennel Club (AKC) breed standards: small (<9 kg), medium (9.5–22.5 kg), large (23–45 kg), and giant (>45 kg) [35]. The defined size of the breeds was used to divide the dogs into age groups: puppy (up to 5 months), juvenile (6–12 months), young adult (1–2 years), mature adult (2–6 years), senior (7–11 years), and geriatric (12–18+ years) [36]. The available individual characteristics, including the breed, age, and occurrence of cryptorchidism of the affected dogs, are given in Table 2.

### 4.4. Histopathology

**Figure 3:**
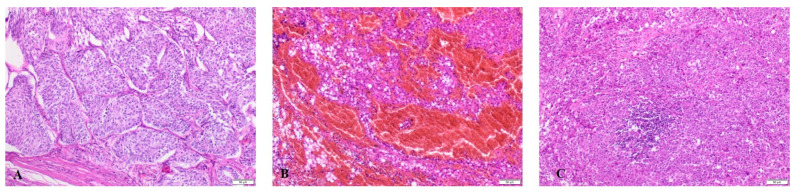
Histopathological evaluations of tumours in canine testes: (**A**) Sertoli cell tumour (SCT), (**B**) Leydig cell tumour (LCT), (**C**) Seminoma (SEM). All tissues were haematoxylin–eosin (HE) stained (×100).

At first, the post-operative tumour tissue was evaluated histopathologically. The collected samples were routinely fixed with 10% buffered formalin (pH 7.2), passed through increasing concentrations of alcoholic solutions to acetone and xylene, and embedded in paraffin blocks. For each tumour, a 4-μm-thick section was stained with haematoxylin and eosin (H&E) and examined under a light microscope coupled with a digital camera (Olympus BX43, Olympus SC100, Tokyo, Japan) in accordance with the WHO histological recommendations [37]. The histopathological evaluations of selected testicular tumours are presented in Figure 3.

### 4.5. Molecular Analysis

The DNeasy Blood & Tissue Kit (Qiagen, Hilden, Germany) was used to extract DNA from tissue and blood according to the manufacturer’s instructions. The concentration of isolated DNA samples was measured using a NanoDrop™ One/OneC Microvolume UV-Vis spectrophotometer (Thermo Scientific, Waltham, MA, USA), and quality was assessed by electrophoretic separation in 1.5% agarose gel (multiSUB™ Maxi, Cleaver Scientific Ltd., Rugby, UK).

To obtain the entire mitochondrial genome sequence for each sample, selective amplification was performed using two pairs of primers obtained from the literature: F1418 and R11041, ~9.5kb PCR product, F9190 and R2382, and ~9.8kb PCR product [38]. Next, full coverage of mtDNA for each sample was obtained after amplification of two long-range PCR products obtained using KAPA HiFi PCR Kit reagents (KAPA Biosystems, Wilmington, NC, USA). The PCR DNA template mix (~1 ng) was used as an input, and an Illumina shotgun library was constructed using the Nextera XT Kit (Illumina, San Diego, CA, USA) following the manufacturer’s instructions. The obtained library sample was sequenced on an Illumina MiSeq sequencer (Illumina, San Diego, CA, USA) using a 600-cycle kit (v3) in a paired-end mode targeting at least 500× coverage. The detailed sequencing information is presented in Table S8.

The mtDNA sequences were analysed in comparison with the reference sequence (GenBank accession No. U96639) [39] using the Unipro UGENE bioinformatic program (v.34.0) [40] to identify changes in nucleotide sequences. A mutation was determined when mtDNA alteration was found only in one out of three tested samples, and a polymorphism was observed when a nucleotide change was identified in all three tested samples compared to the reference sequence [41]. Thereafter, bioinformatics tools, such as ExPASy Server [42], SIFT (sorting intolerant from tolerant) [43], and SOPMA (Self-Optimised Prediction Method with Alignment) [44,45], were used for detailed physicochemical and structural protein analyses. The identified variants in DNA and proteins were described according to the guidelines of the Human Genome Variation Society (HGVS) [46].

## 5. Conclusions

The molecular landscape of tumourigenesis is complex and enormously diversified. The present analyses confirmed the numerosity of changes in mtDNA, including SNPs, indels, indel mutations, and heteroplasmy. Based on the obtained results, mtDNA variants common for all samples, as well as additional alterations specific to benign neoplasms were indicated. As predicted, the highest diversity was noted in the malignant testicular tumours.

The polymorphisms commonly observed in the tested samples were identified in protein-coding genes, especially in proteins encoded in complexes IV, I, and V of OXPHOS. However, the vast majority of these changes were silent mutations. Afterwards, mitochondrial variants were found preferentially in the non-coding D-loop, SNPs were more frequent in the HVI region, and heteroplasmy was detected in the VNTR region. Interestingly, also polymorphisms were noted in 10 out of 22 tRNA genes in the benign and malignant tumours. The mitochondrial changes in tRNAs were mainly observed in the DHU arm/loop or in the TΨC arm/loop. The described tRNA variants may disrupt efficient protein synthesis, albeit their biological consequences may be assessed only in additional detailed research.

So far, investigations of TTs consisting of genetic analyses, especially those based on mtDNA, have been very limited. To the best of the authors’ knowledge, this is the first study to provide new insight into the molecular background of the well-known issue of TTs and may serve as a starting point for further extended research.

## Figures and Tables

**Table 1:** List of common polymorphisms present in all tested dogs and additional changes occurring only in dogs with benign TTs.

**List of Common Polymorphisms Occurring in Each Dog with TT**					
**Gene/Region**	**Reference Sequence**	**Sequence Variant**	**Codon Change/Position in tRNA**	**Amino Acid Change/tRNA Region**	**SIFT ^1^ for Non-Synonymous Changes**
*tRNA* ^*Leu* (*UUR*)^	m.2678	m.2678_2679insG	8_9	between the acceptor stem and DHU stem	-
	m.2683G	m.2683G>A	13	DHU arm	
*COX1*	m.5367C	m.5367C>T	CTG → TTG	p.Leu7=	
	m.5444T	m.5444T>C	GCT → GCC	p.Ala32=	
	m.6065A	m.6065A>G	GGA → GGG	p.Gly239=	
*ATP6*	m.8368C	m.8368C>T	CTC → CTT	p.Leu135=	
*COX3*	m.8807G	m.8807G>A	TGC → TAC	p.Cys55Tyr	tolerant
*ND4L*	m.9911_9912	m.9911_9912insGT	ATG → GTG	p.Met1Val	intolerant
*ND5*	m.13299T	m.13299T>A	TCA → ACA	p.Ser508Thr	tolerant
D-loop	m.15639T	m.15639T>G	-	-	-
	m.15814C	m.15814C>T			
**List of Additional Polymorphisms Occurring Only in Dogs with Benign TT**					
**Gene/Region**	**Reference Sequence**	**Sequence Variant**	**Codon Change/Position in tRNA**	**Amino Acid Change/tRNA Region**	**SIFT for Non-Synonymous Changes**
*ND1*	m.2962C	m.2962C>T	ATC → ATT	p.Ile=	-
	m.3196T	m.3196T>C	CTT → CTC	p.Leu=	
*ATP6*	m.8281T	m.8281T>C	ATT → ATC	p.Ile=	
*ND4*	m.10992G	m.10992G>A	TTG → TTA	p.Leu=	
*CYB*	m.15214G	m.15214G>A	TTG → TTA	p.Leu=	

^1^ SIFT—sorting intolerant from tolerant.

**Table 2:** List of dogs diagnosed with TTs, histopathological assessment, and characteristics of individuals.

Number	Breed/ Crossbreed	Sample Number	Age of Dog	Size of the Dog According to AKC ^1^	Age Group	Abnormalities of Testes	Tumour Type ^2^	Malignant/Benign
1	Crossbreed	T163	8	M	senior	unilateral	SEM	malignant
2		T138	10			bilateral		
3		T087	11			unilateral + cryptorchidism	SCT	benign
4		T015	12		geriatric	unilateral	LCT	malignant
5	German Shepherd	T104	10	L	senior	unilateral + cryptorchidism	SCT	
6		T121				unilateral	LCT	benign
7		T149	11					
8		T156	14		geriatric		SCT	malignant
9	Giant Schnauzer	T139	14					
10	Golden Retriever	T195	9		senior		LCT	benign
11	Labrador Retriever	T105	8				SEM	malignant
12	Miniature Schnauzer	T141	14	S	geriatric		LCT	benign
13	Poodle (Miniature)	T153	15				SEM	malignant
14	Prague Ratter	T137	14			unilateral + cryptorchidism		
15	Standard Schnauzer	T066	10	M	senior			

^1^ The size of the dogs was determined according to the animal’s body weight and according to the American Kennel Club (AKC) breed standards; viz (S) small (<9 kg), (M) medium (9.5–22.5 kg), (L) large (23–45 kg); ^2^ SEM-seminoma; SCT-Sertoli cell tumour; LCT-Leydig cell tumour.

## Data Availability

The datasets used and analysed in the current study are available from the corresponding author upon reasonable request.

## Supplementary Materials

The supporting information can be downloaded at https://www.mdpi.com/article/10.3390/ijms25189944/s1.
