# Supplementary material for: Whole Mitochondrial Genome Sequencing Analysis of Canine Testicular Tumours
Source: Int J Mol Sci. 2024 Sep 14;25(18):9944. doi: 10.3390/ijms25189944 (PMC11432695; doi:10.3390/ijms25189944)
Supplement: Supplementary file 1 [file ijms-25-09944-s001.zip › ijms-3182679-supplementary.pdf]

Whole mitochondrial genome sequencing analysis of canine testicular tumours

Angelika Tkaczyk-Wlizło[1], Krzysztof Kowal[1], Anna Śmiech [2], Brygida Ślaska[1*]

[1] Institute of Biological Bases of Animal Production, University of Life Sciences in Lublin, Akademicka 13 St., 20-950 Lublin, Poland.

[2] Department of Pathomorphology and Forensic Medicine, Faculty of Veterinary Medicine, University of Life Sciences in Lublin, Głęboka 30 St, 20-612 Lublin, Poland.

* Corresponding author. Tel.: +48 814456898, e-mail address: brygida.slaska@up.lublin.pl

Supporting Information

Supplementary items:

Table S1. List of polymorphisms detected in blood, tumour, and healthy tissue collected from dogs with testicular tumours generated using Unipro uGene.

Table S2. Heteroplasmy and polymorphisms found in the VNTR region of the D-loop between 16,130 and 16,430 nucleotides.

Table S3. Total number of polymorphisms, mutations, heteroplasmy, and indels found in the analysed samples.

Table S4. Number and percentage of polymorphisms identified in malignant and benign testicular tumours.

Table S5. List of mutations and heteroplasmy found in genes and mutations in the non-coding D-loop region present in dogs with TTs.

Table S6. List of polymorphisms identified in tRNA genes in samples collected from dogs with TTs. Localisation of tRNA variants were determined using the Canis mitoSNP tool (Kowal et al. 2023).

Table S7. List of non-synonymous changes identified in protein-coding genes and their protein data from SIFT and SOPMA.

Table S8. Detailed information about sequencing data generated using the Illumina MiSeq sequencer.

Table S1. List of polymorphisms detected in blood, tumour, and healthy tissue collected from dogs with testicular tumours generated using Unipro uGene.

Positon	*GENE/ REGION*	S_REF	T015	T066	T087	T104	T105	T121	T137	T138	T139	T141	T149	T153	T156	T163	T195	Position	*GENE/ REGION*	S_REF	T015	T066	T087	T104	T105	T121	T137	T138	T139	T141	T149	T153	T156	T163	T195
16	*tRNA* ^{Phe}	T	C	C	.	.	.	.	.	.	C	C	.	.	.	.	.	3106	*ND1*	A	.	.	.	.	.	.	.	G	.	.	.	.	.	.	.
165	*12s rRNA*	T	.	.	.	.	.	.	.	C	.	.	.	.	.	.	.	3196		T	C	C	C	C	C	C	.	C	C	C	C	C	.	C	C
381		T	A	A	.	.	.	.	.	.	A	A	.	.	.	.	.	3406		C	T	T	.	.	.	.	.	T	T	T	.	.	.	.	.
445		A	.	G	.	.	.	.	.	.	.	G	.	.	.	.	.	3469		G	A	A	.	.	.	.	.	.	A	A	.	.	.	.	.
463		T	.	.	.	.	.	.	.	.	.	.	.	C	.	.	.	3494		T	.	.	.	.	.	.	.	.	C	.	.	.	.	.	.
557		A	G	.	.	.	.	.	.	.	G	.	.	.	.	.	.	3598		G	A	A	.	.	.	.	.	.	A	A	.	.	.	.	.
571		T	.	.	.	.	.	.	.	.	.	C	.	.	.	.	.	4135	*ND2*	C	.	.	.	.	.	.	.	.	.	.	.	T	.	.	.
947		C	.	.	.	.	.	.	.	T	.	.	.	.	.	.	.	4169		A	.	.	.	.	.	.	.	.	.	.	.	G	.	.	.
1046	*tRNA* ^{Val}	G	A	.	.	.	.	.	.	.	.	.	.	.	.	.	.	4204		G	.	A	.	.	.	.	.	.	.	A	.	.	.	.	.
1204	*16s rRNA*	T	C	.	.	.	.	.	.	.	C	.	.	.	.	.	.	4234		C	T	.	.	.	.	.	.	.	T	.	.	.	.	.	.
1351		A	.	.	.	G	G	G	.	.	.	.	.	.	.	G	G	4277		A	.	G	.	.	.	.	.	.	.	G	.	.	.	.	.
1454		G	A	A	.	.	.	.	.	A	A	A	.	.	.	.	.	4303		A	.	.	.	G	G	G	.	.	.	.	.	.	.	G	.
1493		-	.	A	.	.	.	.	.	.	A	A	.	.	.	.	.	4390		T	.	C	.	.	.	.	.	.	.	C	.	.	.	.	.
1662		C	.	.	.	.	.	.	.	.	.	.	.	T	.	.	.	4503		A	G	.	.	.	.	.	.	.	G	.	.	.	.	.	.
1709		G	A	.	.	.	.	.	.	.	A	.	.	.	.	.	.	4517		G	A	A	.	.	.	.	.	.	A	A	.	.	.	.	.
1748		T	C	.	.	.	.	.	.	.	C	.	.	.	.	.	.	4572		T	.	.	.	.	.	.	.	.	.	.	.	C	.	.	.
1756		C	T	T	.	.	.	.	.	.	T	T	.	.	.	.	.	4646		T	.	C	.	.	.	.	.	.	.	C	.	.	.	.	.
2185		T	.	C	.	.	.	.	.	.	.	C	.	.	.	.	.	4940		T	.	.	.	C	C	C	.	.	.	.	.	.	.	C	C
2232		A	G	G	.	.	.	.	.	.	G	G	.	.	.	.	.	5009	*tRNA* ^{Trp}	C	T	T	.	.	.	.	.	.	T	T	.	.	.	.	.
2678	*tRNA* $^{Leu\ (UUR)}$	-	insG															5126	*tRNA* ^{Asn}	C	.	.	.	.	.	.	.	T	.	.	.	.	.	.	.
2683		G	A	A	A	A	A	A	A	A	A	A	A	A	A	A	A	5367	*COX1*	C	T	T	T	T	T	T	T	T	T	T	T	T	T	T	T
2800	*ND1*	C	.	.	.	.	.	.	.	T	.	.	.	.	.	.	.	5444		T	C	C	C	C	C	C	C	C	C	C	C	C	C	C	C
2812		C	.	T	.	.	.	.	.	.	.	T	.	.	.	.	.	5624		G	A	.	.	.	.	.	.	.	A	.	.	.	.	.	.
2833		C	.	.	C	.	.	.	.	.	.	.	C	.	.	.	.	5855		C	.	.	.	.	.	.	.	.	.	.	.	T	.	.	.
2962		C	T	T	T	T	T	T	.	T	T	T	T	T	.	T	T	5906		A	.	G	.	.	.	.	.	.	.	.	.	.	.	.	.
3028		A	.	C	.	.	.	.	.	.	.	C	.	.	.	.	.	6065		A	G	G	G	G	G	G	G	G	G	G	G	G	G	G	G

Position	*GENE/ REGION*	S_REF	T015	T066	T087	T104	T105	T121	T137	T138	T139	T141	T149	T153	T156	T163	T195
6236	*COX1*	A	.	.	.	.	.	.	.	G	.	.	.	.	.	.	.
6257		G	A	A	.	.	.	.	.	.	A	A	.	.	.	.	.
6302		A	.	.	G	.	.	.	.	.	.	.	G	.	.	.	.
6401		C	.	.	.	T	T	T	.	.	.	.	.	.	.	T	T
6407		A	.	.	.	.	.	.	.	G	.	.	.	.	.	.	.
6470		G	A	A	.	.	.	.	.	.	A	A	.	.	.	.	.
6518		G	.	.	.	.	.	.	.	.	.	.	.	A	.	.	.
6554		T	.	.	.	C	C	C	.	.	.	.	.	.	.	C	C
6683		T	.	.	C	.	.	.	.	.	.	.	C	.	.	.	.
6711		T	.	.	.	.	.	.	.	.	A	.	.	.	.	.	.
6740		G	.	.	.	.	.	.	.	.	A	.	.	.	.	.	.
6764		C	.	T	.	.	.	.	.	.	.	T	.	.	.	.	.
6836		T	.	C	.	.	.	.	.	.	.	.	.	.	.	.	.
6863		C	.	.	.	.	.	.	.	.	T	.	.	.	.	.	.
6967	*tRNA* Asp	A	.	.	.	.	.	.	.	.	.	.	.	G	.	.	.
7014		T	.	C	.	.	.	.	.	.	.	C	.	.	.	.	.
7058	*COX2*	T	C	.	.	.	.	.	.	.	C	.	.	.	.	.	.
7112		A	G	.	.	.	.	.	.	.	.	.	.	.	.	.	.
7186		C	A	.	.	.	.	.	.	.	.	.	.	.	.	.	.
7363		T	C	.	.	.	.	.	.	.	.	.	.	.	.	.	.
7383		A	.	.	G	.	.	.	.	.	.	.	G	.	.	.	.
7426		A	.	.	G	.	.	.	.	.	.	.	.	.	.	.	.
7593		T	.	.	.	C	C	C	.	.	.	.	.	.	.	C	C
7914	*ATP8*	A	.	.	.	.	.	.	.	G	.	.	.	.	.	.	.
7923		T	.	.	.	.	.	.	.	.	.	.	.	C	.	.	.
8048	*ATP6*	A	.	.	.	.	.	.	.	G	.	.	.	.	.	.	.

Position	*GENE/ REGION*	S_REF	T010	T066	T087	T104	T105	T121	T137	T138	T139	T141	T149	T153	T156	T163	T195
8101	*ATP6*	G	.	A	.	.	.	.	.	.		A	.	.	.	.	.
8221		A	C	C	.	.	.	.	.	.	C	C	.	.	.	.	.
8225		T	C	C	.	.	.	.	.	.	C	C	.	.	.	.	.
8242		G	.	.	A	.	.	.	.	.	.	.	A	.	.	.	.
8281		T	C	C	C	C	C	C	.	C	C	C	C	C	.	C	C
8323		A	G	G	.	.	.	.	.	G	G	G	.	.	.	.	.
8368		C	T	T	T	T	T	T	T	T	T	T	T	T	T	T	T
8411		T	.	.	.	.	.	.	.	C		.	.	.	.	.	.
8425		G	A	A	.	.	.	.	.	.	A	A	.	.	.	.	.
8536		C	.	.	.	.	.	.	T	.	.	.	.	.	T	.	.
8569		A	.	G	.	.	.	.	.	.	.	G	.	.	.	.	.
8703	*COX3*	G	A	A	.	.	.	.	.	A	A	A	.	.	.	.	.
8736		T	.	C	.	.	.	.	.	.	.	C	.	.	.	.	.
8760		A	G	G	.	.	.	.	.	.	G	G	.	.	.	.	.
8764		G	T	T	.	.	.	.	.	T	T	T	.	.	.	.	.
8807		G	A	A	A	A	A	A	A	A	A	A	A	A	A	A	A
8817		A	.	G	.	.	.	.	.	.	.	G	.	.	.	.	.
8877		A	G	G	.	.	.	.	.	.	G	G	.	.	.	.	.
8991		A	G	G	.	.	.	.	.	.	G	G	.	.	.	.	.
9219		A	.	G	.	.	.	.	.	.	.	G	.	.	.	.	.
9222		C	T	.	.	.	.	.	.	.	T	.	.	.	.	.	.
9708	*ND3*	C	T		.	.	.	.	.	.	T	.	.	.	.	.	.
9825		G	.	A	.	.	.	.	.	.	.	A	.	.	.	.	.
9838		G	.	.	.	.	.	.	.	.	.	.	.	A	.	.	.
9865	*tRNA* Arg	-	A	.	.	.	.	.	.	.	A	.	.	.	.	.	.
9896		T	.	.	C	.	.	.	.	.	.	.	C	.	.	.	.

Position	GENE/ REGION	S_REF	T015	T066	T087	T104	T105	T121	T137	T138	T139	T141	T149	T153	T156	T163	T195	Position	GENE/ REGION	S_REF	T010	T066	T087	T104	T105	T121	T137	T138	T139	T141	T149	T153	T156	T163	T195
9911	*ND4L*	-	insTG															11657	*tRNA* *Ser (AGY)*	C	.	.	A	.	.	.	.	.	.	.	A	.	.	.	.
9988		A	.	.	.	.	.	.	.	.	.	.	.	.	.	T	.	11728	*tRNA* *Leu (CUN)*	A	.	.	.	.	.	G	.	.	.	.	.	.	.	.	.
10165		C	.	.	.	.	.	.	T	.	.	.	.	.	T	.	.	11813	*ND5*	A	.	.	G	.	.	.	.	.	.	.	G	.	.	.	.
10257	*ND4*	G	.	A	.	.	.	.	.	.	.	A	.	.	.	.	.	11839		T	C	.	.	.	.	.	.	.	.	.	.	.	.	.	.
10346		C	.	.	.	.	.	.	.	.	.	.	.	T	.	.	.	11948		A	.	G	.	.	.	.	.	.	.	G	.	.	.	.	.
10354		T	.	.	.	.	.	.	.	C	.	.	.	.	.	.	.	11959		C	.	.	.	.	.	.	.	.	T	.	.	.	.	.	.
10404		C	T	T	.	.	.	.	.	.	T	T	.	.	.	.	.	11963		C	T	T	.	.	.	.	.	.	T	T	.	.	.	.	.
10440		T	.	C	.	.	.	.	.	.	.	C	.	.	.	.	.	12063		G	.	.	.	.	.	.	.	.	.	.	.	A	.	.	.
10533		A	T	T	.	.	.	.	.	.	T	T	.	.	.	.	.	12122		C	T	.	.	.	.	.	.	.	T	.	.	.	.	.	.
10542		A	.	G	.	.	.	.	.	.	.	G	.	.	.	.	.	12200		C	.	.	.	.	T	.	.	.	.	.	.	.	.	T	.
10611		A	.	.	.	T	T	T	.	.	.	.	.	.		T	T	12272		T	C	.	.	.	.	.	.	.	C	.	.	.	.	.	.
10613		A	.	.	.	.	.	.	.	.	.	.	.	G	.	.	.	12330		A	G	.	.	.	.	.	.	.	G	.	.	.	.	.	.
10776		T	C	.	.	.	.	.	.	.	C	.	.	.	.	.	.	12346		T	.	.	.	.	.	.	.	.	A	.	.	.	.	.	.
10785		A	G	.	.	.	.	.	.	.	G	.	.	.	.	.	.	12401		T	.	.	.	.	.	.	.	.		.	.	C	.	.	.
10863		A	G	.	.	.	.	.	.	.	.	.	.	.	.	.	.	12636		T	C	.	.	.	.	.	.	.	C	.	.	.	.	.	.
10917		G	A	A	.	.	.	.	.	A	A	A	.	.	.	.	.	12665		T	.	C	.	.	.	.	.	.	.	C	.	.	.	.	.
10992		G	A	A	A	A	A	A	.	A	A	A	A	.	A	A	A	12788		T	C	C	.	.	.	.	.	C	C	C	.	.	.	.	.
11100		G	A	.	.	.	.	.	.	.	.	.	.	.	.	.	.	12813		G	A	A	.	.	.	.	.	.	A	A	.	.	.	.	.
11172		A	.	.	.	.	.	.	.	.	.	.	.	G	.	.	.	13112		G	A	.	.	.	.	.	.	.	.		.	.	.	.	.
11176		C	.	T	.	.	.	.	.	.	.	T	.	.	.	.	.	13202		T	.	.	.	.	.	.	.	C	.		.	.	.	.	.
11247		A	.	.	.	.	.	.	.	.	G	.	.	.	.	.	.	13261		C	T	T	.	.	.	.	.		T	T	.	.	.	.	.
11250		T	C	C	.	.	.	.	.	.	C	C	.	.	.	.	.	13299		T	A	A	A	A	A	A	A	A	A	A	A	A	A	A	A
11322		T	C	.	.	.	.	.	.	.	C	.	.	.	.	.	.	13427		T	.	.	.	.	.	.	.	C	.	.	.	.	.	.	.
11400		T	C	.	.	.	.	.	.	.	C	.	.	.	.	.	.	13459		T	.	.	.	.	.	.	.	C	.	.	.	.	.	.	.
11402		T	C	C	.	.	.	.	.	.	C	C	.	.	.	.	.	13594	*ND5/ND6***	G	.	A	.	.	.	.	.	.		A	.	.	.	.	.
11572		A	C	.	.	.	.	.	.	.	C	.	.	.	.	.	.	13618	*ND6*	A	G	G	.	.	.	.	.	.	G	G	.	.	.	.	.

Position	*GENE/ REGION*	S_REF	T015	T066	T087	T104	T105	T121	T137	T138	T139	T141	T149	T153	T156	T163	T195	Position	*GENE/ REGION*	S_REF	T015	T066	T087	T104	T105	T121	T137	T138	T139	T141	T149	T153	T156	T163	T195
13660	*ND6*	C	T	T	.	.	.	.	.	.	T	T	.	.	.	.	.	15526	D-LOOP HVI	C	T	T	.	.	.	.	.	.	T	T	.	.	.	.	.
13708		C	T	T	.	.	.	.	.	.	T	T	.	.	.	.	.	15553		A	.	G	.	.	.	.	.	.	.	.	.	.	.	.	.
13777		G	A	A	.	.	.	.	.	.	A	A	.	.	.	.	.	15595		C	.	T	.	.	.	.	.	.	.	T	.	.	.	.	.
13791		T	.	.	.	.	.	.	.	.	C	.	.	.	.	.	.	15611		T	.	.	.	.	.	.	.	.	C	.	.	.	.	.	.
13802		G	.	.	.	.	.	.	.	.	.	.	.	A	.	.	.	15612		T	.	C	.	.	.	.	.	.	.	C	.	.	.	.	.
13864		C	.	.	.	.	.	.	.	T	.	.	.	.	.	.	.	15620		T	.	.	.	C	C	C	.	.	.	.	.	.	.	C	C
14329	*CYTB*	A	.	.	.	.	.	.	.	G	.	.	.	.	.	.	.	15621		C	T	.	.	.	.	.	.	.	.	.	.	.	.	.	.
14383		C	.	.	.	.	.	.	.	G	.	.	.	.	.	.	.	15627		A	.	.	G	G	G	G	.	.	G	.	G	G		G	G
14425		T	.	.	.	.	.	.	.	C	.	.	.	.	.	.	.	15632		C	.	T	T	.	.	.	.	.	.	T	T	.	.	.	.
14467		C	.	.	.	.	.	.	.	T	.	.	.	.	.	.	.	15639		T	G	G	A	A	A	A	A	A	G	G	A	A	A	A	A
14474		G	.	.	.	.	.	.	A	.	.	.	.	.	A	.	.	15643		A	.	G	.	.	.	.	.	.	.	G	.	.	.	.	.
14534		G	.	.	A	.	.	.	.	.	.	.	A	.	.	.	.	15650		T	C	.	.	.	.	.	.	.	C	.	.	.	.	.	.
14608		A	G	.	.	.	.	.	.	.	G	.	.	.	.	.	.	15652		G	.	A	.	.	.	.	.	A	.	A	.	.	.	.	.
14647		T	C	.	.	.	.	.	.	.	C	.	.	.	.	.	.	15710		C	.	.	.	.	.	.	.	.	T		.	.	.	.	.
14671		G	.	A	.	.	.	.	.	.	.	A	.	.	.	.	.	15750		C	.	.	T	.	.	.	.	.	.	.	T	.	.	.	.
14692		G	A	.	.	.	.	.	.	.	A	.	.	.	.	.	.	15800		T	C	C	.	.	.	.	.	.	C	C	.	.	.	.	.
14930		T	.	C	.	.	.	.	.	.	.	C	.	.	.	.	.	15814		C	T	T	T	T	T	T	T	T	T	T	T	T	T	T	T
14977		T	.	.	.	C	C	C	.	.	.	.	.	.	.	C	C	15815		T	.	C	.	.	.	.	.	.	.	C	.	.	.	.	.
14998		A	.	.	.	.	.	.	.	G	.	.	.	.	.	.	.	15912		C	T	T	.	.	.	.	.	.	T	T	.	.	.	.	.
15185		T	C	.	.	.	.	.	.	.	C	.	.	.	.	.	.	15938		G	-	.	.	.	.	.	.	.	-	.	.	.	.	.	.
15213		G	A	A	A	A	A	A	.	A	A	A	A	A	.	A	A	15955		C	T	T	.	T	T	T	.	.	T	T	.	.	.	T	.
15220		T	.	.	.	.	.	.	.	.	.	.	.	A	.	.	.	16003		A	G	G	.	.	.	.	.	.	G	G	.	.	.	.	.
15372	*tRNA Thr*	G	.	.	.	.	.	.	.	.	A	.	.	.	.	.	.	16025		T	.	.	C	.	.	.	C	.	.	.	C	.	C	.	.
15435	*tRNA Pro*	G	A	A	.	.	.	.	.	.	A	A	.	.	.	.	.	16083		A	.	G	.	.	.	.	.	.	.	G	.	.	.	.	.
15475	D-LOOP HVI	T	.	.	C	.	.	.	.	.	.	.	C	.	.	.	.	16128		G	.	A	.	.	.	.	.	.	.	A	.	.	.	.	.
15483		C	.	.	.	.	.	.	.	.	.	.	.	T	.	.	.	16148	*D-loop (VNTR)*	A	.	.	G	.	G	.	.	.	.	.	.	G		G	G
15508		C	T	.	.	.	.	.	.	.	T	.	.	.	.	.	.	16158		A	.	.	.	.	.	.	.	.	.	.	.	G	.	.	.

Position	*GENE/ REGION*	S_REF	T015	T066	T087	T104	T105	T121	T137	T138	T139	T141	T149	T153	T156	T163	T195
16168	*D-loop (VNTR)*	A	.	.	G	.	.	.	.	.	.	.	.	.	.	G	G
16198		G	.	.	.	.	.	.	.	.	.	.	.	.	.	.	A
16248		A	.	.	.	G	.	.	.	.	.	.	.	.	.	.	.
16258		G	.	A	.	.	.	.	.	.	.	.	.	.	.	.	.
16268		A	.	.	.	G	.	.	.	.	.	.	.	.	.	.	.
16288		A	.	.	.	G	.	G	.	.	.	.	.	.	.	.	.
16298		A	.	.	.	G	.	.	.	.	.	.	.	.	.	.	.
16318		A	.	.	.	G	.	G	.	.	.	.	.	.	.	.	G
16358		A	.	.	.	.	.	.	-[1]	.	.	.	.	.	.	G	.
16368		G	A	A	.	.	.	.	.	.	.	.	.	.	.	A	.
16378		G	A	.	.	.	A	.	.	.	.	.	.	.	.	.	.
16388		A	.	.	G	G	G	.	.	.	.	.	.	.	.	.	G
16398		A	G	G	G	G	.	G	.	.	.	G	.	G	.	G	G
16408		G	.	.	A	.	.	.	.	.	.	.	.	.	.	.	.
16408		.	.	.	.	.	.	.	.	.	.	.	-[2]	.	.	.	.
16418		A	G	G	G	G	G	G	.	-[3]	G	.	.	G	.	G	G
16421		.	.	.	.	.	.	.	.	-[3]	.	.	.	.	.	.	.
16431	D-LOOP HVII	C	T	T	.	.	.	.	.	.	T	.	.	.	.	.	.
16439		T	C	C	.	.	.	.	.	.	C	C	.	.	.	.	.
16663		-	.	.	.	insCC			.	.	.	.	.	.	.	insCC	
16671		T	C	.	.	.	.	.	.	.	C		.	.		.	.
16672		C	.	T	.	T	.	T	.	.	.	T	.	.	T	.	.
16705		C	T	.	.	.	.	.	.	.	.	.	.	.	.	.	.

- deletion; - [1] – m.16358delGTACACGTGC; - [2] – m.16408delGTACACGTAC; - [3] – m.16418del GTACACGTAC; ** - the polymorphisms occurred in a region spanning ND5 and ND6 genes; . .– the variant is the same as in the reference sequence.

Table S2. Heteroplasmy and polymorphisms found in the VNTR region of the D-loop between 16,130 and 16,430 nucleotides.

Sample/ Position	16138	16148	16158	16168	16178	16188	16198	16208	16218	16228	16238	16248	16258	16268	16278	16288	16298	16308	16318	16326	16328	16338	16348	16356	16358	16368	16378	16388	16398	16408	16418
**S_REF**	A	A	A	A	A	G	G	A	G	G	G	A	G	A	G	A	A	G	A	G	G	G	G	G	A	G	G	A	A	G	A
**T015B***	.	.	.	R	.	R	R	.	.	A	.	R	.	R	.	R	.	R	R	.	.	.	.	.	.	A	A	.	.	.	G
**T015T***	.	.	.	R	R	R	R	.	.	A	.	R	.	R	.	R	.	R	R	.	.	.	.	.	.	A	A	.	.	.	G
**T015H***	.	.	.	R	R	R	R	.	.	R	.	R	.	R	.	R	.	R	R	.	.	.	.	.	.	A	A	.	.	.	G
**T066B**	.	.	.	R	R	R	R	.	.	.	R	.	A	.	R	.	.	.	R	.	R	R	A	.	.	A	A	G	G	.	G
**T066T**	.	.	.	.	R	R	R	.	R	.	R	.	A	.	R	.	.	R	R	.	R	R	R	.	.	A	R	R	G	.	G
**T066H**	.	.	.	R	R	R	R	.	R	.	R	.	A	.	R	.	.	R	R	.	R	R	R	.	.	A	A	R	G	.	G
**T087B**	.	G	.	G	.	R	R	.	.	A	.	.	.	.	.	R	R	.	R	.	.	.	.	.	R	R	.	G	G	A	G
**T087T**	.	G	.	G	.	.	A	.	.	A	.	.	.	R	.	R	R	.	R	.	.	.	.	.	R	R	.	G	G	A	G
**T087H**	.	G	.	G	.	R	R	.	.	R	.	.	.	.	.	.	R	.	R	.	.	.	.	.	R	R	.	G	G	A	G
**T104B**	.	.	R	R	.	R	R	R	.	.	.	G	.	G	.	G	G	.	G	.	.	.	.	.	R	R	.	G	G	.	G
**T104T**	.	.	G	R	.	R	R	R	.	.	.	G	.	G	.	G	G	.	G	.	.	.	.	.	G	R	.	G	G	.	G
**T104H**	.	.	R	R	.	R	R	R	.	.	.	G	.	G	.	G	G	.	G	.	.	.	.	.	R	R	.	G	G	.	G
**T105B**	.	G	.	R	R	R	.	G	.	.	.	.	.	.	.	R	R	.	.	.	.	.	.	R	.	.	A	G	.	.	G
**T105T**	.	G	.	R	R	R	.	R	.	.	.	.	.	.	.	R	R	.	.	.	.	R	.	R	.	.	A	G	.	.	G
**T105H**	.	G	.	R	R	R	.	G	.	.	.	.	.	.	.	R	R	.	.	.	.	R	.	R	.	.	A	G	.	.	G
**T121B**	.	.	.	.	R	R	.	R	.	.	.	R	.	R	.	G	R	.	G	.	.	R	.	.	R	R	.	G	G	.	G
**T121T**	.	.	.	.	R	R	.	R	.	.	.	R	.	R	.	G	R	.	G	.	.	.	.	.	R	R	.	-	G	.	G
**T121H**	.	.	.	.	R	R	.	R	.	.	.	R	.	R	.	G	R	.	G	.	.	.	.	.	R	R	.	G	G	.	G
**T137B**	.	G	.	G	.	R	.	.	R	R	.	.	.	.	.	R	R	.	.	.	.	R	.	.	-[1]	.	.	.	.	.	.
**T137T**	.	.	G	.	R	A	.	.	R	R	R	.	.	.	R	.	.	.	.	.	.	R	R	.	-[1]	.	.	.	.	.	.
**T137H**	.	G	.	G	.	R	.	.	.	R	R	.	.	.	.	R	.	.	.	.	.	A	.	.	-[1]	.	.	.	.	.	.
**T138B**	.	R	R	.	R	.	.	R	R	R	R	R	R	R	.	R	R	.	.	.	.	R	.	.	.	.	.	.	.	.	-[3]

Sample/ Position	16138	16148	16158	16168	16178	16188	16198	16208	16218	16228	16238	16248	16258	16268	16278	16288	16298	16308	16318	16326	16328	16338	16348	16356	16358	16368	16378	16388	16398	16408	16418
**T138T**	.	.	R	.	R	R	R	.	R	R	R	R	R	R	.	.	R	.	.	.	.	A	.	.	.	.	.	.	.	.	-[3]
**T138H**	.	.	R	R	R	R	R	R	R	R	R	R	R	R	.	R	R	.	.	.	.	R	.	.	.	.	.	.	.	.	-[3]
**T139B**	.	.	R	.	R	.	.	R	.	.	.	R	.	R	.	.	.	R	R	.	.	.	.	.	R	.	.	R	R	.	G
**T139T**	R	.	.	R	R	.	.	R	.	.	R	R	.	R	.	.	.	R	R	.	.	.	.	.	R	.	.	R	G	.	G
**T139H**	R	R	R	.	R	R	R	R	R	R	R	R	R	R	R	.	R	R	R	.	.	.	R	.	R	.	.	G	G	.	G
**T141B**	.	.	.	R	R	R	R	.	R	R	.	R	.	.	.	R	R	.	R	.	.	.	R	.	R	R	R	R	G	.	G
**T141T**	.	.	.	R	R	R	R	.	R	R	.	R	.	.	.	R	R	.	R	.	.	.	R	.	R	R	.	R	G	.	-[3]
**T141H**	.	.	.	R	R	R	R	.	R	R	.	R	.	.	.	R	R	.	R	.	.	.	R	.	R	R	R	R	G	.	G
**T149B**	.	R	.	R	R	R	R	.	R	R	R	.	.	.	.	.	.	.	.	.	.	R	.	.	R	.	.	R	R	-[2]	G
**T149T**	.	R	.	R	.	R	R	R	R	R	R	R	.	R	.	.	.	.	.	.	.	R	.	.	R	.	.	R	R	-[2]	G
**T149H**	.	R	.	R	R	R	R	.	R	R	R	.	.	.	.	.	R	.	R	.	.	R	.	.	R	.	.	R	R	-[2]	G
**T153B**	.	G	G	.	.	.	.	R	R	R	R	.	.	.	.	.	.	R	R	.	R	R	R	.	R	R	R	R	G	.	G
**T153T**	.	G	G	.	.	.	.	R	R	R	R	.	.	R	.	R	.	R	.	.	R	R	R	.	R	R	R	-	G	.	G
**T153H**	.	G	G	.	.	.	.	R	R	R	R	.	.	R	.	R	.	R	.	.	R	R	R	.		R	R	R	G	.	G
**T156B**	G	G	G	G	.	.	R	.	.	R	R	.	.	.	.	.	.	.	.	.	R	R	R	.	.	.	.	.	.	.	.
**T156T**	.	.	R	R	R	R	R	.	R	R	R	.	.	.	.	.	.	.	.	.	R	R	R	.	.	.	.	.	.	.	.
**T156H**	G	G	G	G	.	.	R	.	.	R	R	.	.	.	.	.	.	.	R	.	R	R	R	.	.	.	.	.	.	.	.
**T163B**	.	G	.	G	.	.	R	R	R	R	.	.	.	.	.	.	R	R	.	.	.	.	.	.	G	A	.	.	G	.	G
**T163T**	.	G	.	G	.	.	R	R	R	R	.	R	R	.	.	R	.	.	R	.	.	.	R	.	G	A	.	.	G	.	G
**T163H**	.	G	.	G	.	R	R	R	R	R	.	.	.	.	.	.	R	R	.	.	.	.	.	.	G	A	.	.	G	.	G
**T195B**	.	G	R	G	R	R	A	R	.	.	.	.	R	R	.	R	R	.	G	.	R	R	R	.	.	R	.	G	G	.	G
**T195T**	.	G	.	G	G	.	A		.	.	.	.	R	R	.	R	R	.	G	R	R	R	R	.	.	R	.	G	G	.	G
**T195H**	.	G	R	G	R	R	A	R	.	.	.	.	R	R	.	R	R	.	G	R	R	R	R	.	.	R	.	G	G	.	G

*B-blood; T-tumour; H- healthy tissue; - – deletion; - [1] – m.16358delGTACACGTGC; - [2] – m.16408delGTACACGTAC; - [3] – m.16418del GTACACGTAC; . .– the variant is the same as in the reference sequence.

Table S3. Total number of polymorphisms, mutations, heteroplasmy, and indels found in the analysed samples.

Gene/ Region	SNP		Indel	
	**Polymorphisms (*)**	**Mutations (*)**	**Polymorphisms**	**Mutations**
12s rRNA	12			
16s rRNA	27	1	3	1
tRNA *Phe*	4			
tRNA *Val*	1			
tRNA *Leu (UUR)*	15		15	
tRNA *Trp*	4			
tRNA *Asn*	1			
tRNA *Asp*	3			
tRNA *Arg*	2		2	
tRNA *Ser (AGY)*	2			
tRNA *Leu (CUN)*	1			
tRNA *Thr*	1			
tRNA *Pro*	4			
ND1	48			
ND2	28	1		
ND3	5			
ND4L	3		30	
ND4	64	(*1)		
ND5	57			
ND5/6*	2			
ND6	19	(*1)		
COX1	80			
COX2	13			
COX3	45	(*2)		
ATP8	2			
ATP6	55			
CYB	40			
**D-loop, HVI**	104		2	1
**D-loop, VNTR**	61 (*31)	10		2
**D-loop, HVII**	19		10	1
**Sum**	722 (*31)	12 (4)	62	5
**Total**	801 (*35)			

*heteroplasmic site; **the polymorphisms occurred in a region spanning *ND5* and *ND6* genes.

Table S4. Number and percentage of polymorphisms identified in malignant and benign testicular tumours.

Gene/ Region	Total	SNP polymorphisms		Total	Indel polymorphisms	
		Malignant [%]	Bening [%]		Malignant [%]	Bening [%]
12s rRNA	12	9 (75,0%)	3 (25,0%)			
16s rRNA	27	21 (77,7%)	6 (22,3%)	3	2 (66,6%)	1 (33,4%)
tRNA ^{Phe}	4	3 (75,0%)	1 (25,0%)			
tRNA ^{Val}	1	1 (100,0%)				
tRNA $^{Leu\ (UUR)}$	15	10 (66,6%)	5 (33,4%)	15	10 (66,6%)	5 (33,4%)
tRNA ^{Trp}	4	3 (75,0%)	1 (25,0%)			
tRNA ^{Asn}	1	1 (100,0%)				
tRNA ^{Asp}	3	2 (66,6%)	1 (33,4%)			
tRNA ^{Arg}	2		2 (100,0%)	2	2 (100,0%)	
tRNA $^{Ser\ (AGY)}$	2		2(100,0%)			
tRNA $^{Leu\ (CUN)}$	1		1 (100,0%)			
tRNA ^{Thr}	1	1 (100,0%)				
tRNA ^{Pro}	4	3 (75,0%)	1 (25,0%)			
ND1	48	31 (64,6%)	17 (35,4%)			
ND2	28	20 (71,4%)	8 (28,6%)			
ND3	5	4 (80,0%)	1 (20,0%)			
ND4L	3	3 (100,0%)		30	20 (66,6%)	10 (33,4%)
ND4	64	48 (75,0%)	16 (25,0%)			
ND5	57	44 (77,2%)	13 (22,8%)			
ND5/6*	2	1 (50,0%)	1 (50,0%)			
ND6	19	15 (79,0%)	4 (21,0%)			
COX1	80	54 (67,5%)	26 (32,5%)			
COX2	13	8 (61,5%)	5 (38,5%)			
COX3	45	32 (71,1%)	13 (28,9%)			
ATP8	2	2 (100,0%)				
ATP6	55	37 (67,3%)	18 (32,7%)			
CYB	40	29 (72,5%)	11 (27,5%)			
**D-loop, HVI**	104	66 (63,5%)	38 (36,5%)	2	2 (100,0%)	
**D-loop, VNTR**	61	40 (65,6)	21 (34,4%)			
**D-loop, HVII**	19	14 (73,7%)	5 (26,3%)	10	6 (60,0%)	4 (40,0%)
**Sum**	722	502 (69,5%)	220 (30,5%)	62	42 (67,7%)	20 (32,3%)

Table S5. List of mutations and heteroplasmy found in genes and mutations in the non-coding D-loop region present in dogs with TTs.

Gene/ Region	Reference sequence	Sequence variant	Codon change	Amino acid changes
12s rRNA	m.634C	m.634C/T*[1]	-	-
16s rRNA	m.1486A	m.1486delAA		
ND2	m.4303A	m.4303A>G	CTA→CTG	p.Leu159=
COX3	m.9041A	m.9041A/G*	AAC→AGC	p.Asn133Ser
ND4	m.11003G	m.11003G/A*	GGC→GAC	p.Gly268Asp
ND6	m.13995T	m.13995T/C*	TTA→TCA	p.Leu52Ser
**D-loop, HVI**	m.15938G	m.15938delG	-	-
**D-loop, VNTR**	m.16158A	m.16158A>G		
	m.16178A	m.16178A>G		
	m.16188G	m.16188G>A		
	m.16198G	m.16198G>A		
	m.16338G	m.16338G>A		
	m.16348G	m.16348G>A		
	m.16358A	m.16358A>G		
	m.16388A	m.16388A>G		
		m.16388delA		

* - heteroplasmy.

Table S6. List of polymorphisms identified in tRNA genes in samples collected from dogs with TTs. Localisation of tRNA variants were determined using the Canis mitoSNP tool (Kowal et al. 2023).

<table>
<thead>
<tr><th>Gene</th><th>Reference sequence</th><th>Sequence variant</th><th>Codon change/ position in tRNA</th><th>Amino acid change/ tRNA region</th><th>Sample number</th><th>Types of testicular tumours*</th></tr>
</thead>
<tbody>
<tr><td>tRNA ^{Phe}</td><td>m.16T</td><td>m.16T>C</td><td>16</td><td>DHU loop</td><td>T15, T66, T139, T141</td><td>LCT, SEM, SCT</td></tr>
<tr><td rowspan="2">tRNA $^{Leu (UUR)}$</td><td>m.2678</td><td>m.2678_2679insG</td><td>8_9</td><td rowspan="2">DHU arm</td><td rowspan="2">all samples</td><td rowspan="2">LCT, SEM, SCT</td></tr>
<tr><td>m.2683G</td><td>m.2683G>A</td><td>13</td></tr>
<tr><td>tRNA ^{Trp}</td><td>m.5009C</td><td>m.5009C>T</td><td>54</td><td rowspan="2">TΨC loop</td><td>T15, T66, T139, T141</td><td>LCT, SEM, SCT</td></tr>
<tr><td>tRNA ^{Asn}</td><td>m.5126C</td><td>m.5126C>T</td><td>53</td><td>T138</td><td rowspan="2">SEM</td></tr>
<tr><td rowspan="2">tRNA ^{Asp}</td><td>m.6967A</td><td>m.6967A>G</td><td>2</td><td>Acceptor arm</td><td>T153</td></tr>
<tr><td>m.7014T</td><td>m.7014T>C</td><td>49</td><td>TΨC arm</td><td>T66, T141</td><td>SEM, SCT</td></tr>
<tr><td rowspan="2">tRNA ^{Arg}</td><td>m.9865</td><td>m. 9865_9866insA</td><td>24</td><td>DHU arm</td><td>T15, T139</td><td rowspan="3">LCT, SCT</td></tr>
<tr><td>m.9896T</td><td>m.9896T>C</td><td>55</td><td>TΨC loop</td><td rowspan="2">T87, T149</td></tr>
<tr><td>tRNA $^{Ser (AGY)}$</td><td>m.11657C</td><td>m.11657C>A</td><td>10</td><td>Central loop</td></tr>
<tr><td>tRNA $^{Leu (CUN)}$</td><td>m.11728A</td><td>m.11728A>G</td><td>21</td><td>DHU arm</td><td>T121</td><td>LCT</td></tr>
<tr><td>tRNA ^{Thr}</td><td>m.15372G</td><td>m.15372G>A</td><td>50</td><td>TΨC loop</td><td>T139</td><td>SCT</td></tr>
<tr><td>tRNA ^{Pro}</td><td>m.15435G</td><td>m.15435G>A</td><td>23</td><td>Central loop</td><td>T15, T66, T139, T141</td><td>LCT, SEM, SCT</td></tr>
</tbody>
</table>

* Types of testicular tumours: LCT - Leydig cell tumour, SCT – Sertoli cell tumour, SEM – seminoma.

Table S7. List of non-synonymous changes identified in protein-coding genes and their protein data from SIFT and SOPMA.

Gene	Sequence variant	Codon change	Amino acid change	SIFT*	Alpha helix	Extended strand	Beta turn	Random coil
ND2			**Normal**		67,46%	8,28%	1,48%	22,78%
	m.4169A>G	ATC→GTC	p.Ile86Val	tolerant	67,46%	9,76%	1,78%	21,01%
	m.4277A>G	ATT→GTT	p.Ile122Val		68,64%	8,58%	2,07%	20,71%
	m.4503A>G	AAC→AGC	p.Asn197Ser		66,86%	10,65%	1,78%	20,71%
	m.4517G>A	GTT→ATT	p.Val202Ile		68,64%	8,58%	2,07%	20,71%
	m.4572T>C	ATA→ACA	p.Met220Thr		67,46%	9,76%	1,78%	21,01%
COX1			**Normal**		51,61%	13,65%	3,41%	31,33%
	m.6711T>A	TCT→ACT	p.Ser455Thr	intolerant	52,41%	12,25%	3,41%	31,93%
COX2			**Normal**		27,48%	23,42%	4,05%	45,05%
	m.7112A>G	ACA→GCA	p.Thr27Ala	tolerant	29,28%	22,06%	4,06%	44,59%
	m.7383A>G	AAC→AGC	p.Asn117Ser		28,83%	22,07%	4,05%	45,05%
	m.7593T>C	ATA→ACA	p.Met187Thr		29,28%	22,07%	4,05%	44,59%
ATP8			**Normal**		21,88%	25,00%	4,69%	48,44%
	m.7914A>G	ATA→GTA	p.Met38Val	tolerant	21,88%	25,00%	4,69%	48,44%
	m.7923T>C	TCT→CCT	p.Ser41Pro		17,19%	26,56%	1,56%	54,69%
ATP6			**Normal**		63,68%	9,42%	4,48%	22,42%
	m.8048A>G	ATT→GTT	p.Ile28Val	tolerant	61,43%	8,97%	4,48%	25,11%
	m.8411T>C	TTT→CTT	p.Phe150Leu		61,43%	8,97%	4,48%	25,11%
COX3			**Normal**		56,00%	12,00%	3,20%	28,80%
	m.8764G>T	GCC→TCC	p.Ala41Ser	tolerant	58,40%	11,20%	2,40%	28,00%
	m.8807G>A	TGC→TAC	p.Cys55Tyr		58,00%	11,60%	2,80%	27,60%
ND3			**Normal**		54,87%	6,19%	3,54%	35,40%
	m.9838G>A	GAA→AAA	p.Glu115Lys	intolerant	61,06%	6,19%	3,54%	29,20%
ND4L			**Normal**		75,51%	9,18%	2,04%	13,27%
	m.10165C>T	GCT→ GTT	p.Ala67Val	intolerant	75,51%	9,18%	2,04%	13,27%
ND4			**Normal**		59,64%	9,64%	3,14%	27,58%
	m.10346C>T	ACA→ATA	p.Thr49Met	tolerant	60,31%	9,19%	3,81%	26,68%
	m.10354T>C	TCC→CCC	p.Ser52Pro		60,31%	10,54%	3,59%	25,56%
	m.10613A>G	AAT→AGT	p.Asn138Ser	intolerant	60,31%	9,19%	3,81%	26,68%
	m.10917G>A	GGC→GAC	p.Gly240Asp		62,33%	9,64%	3,36%	24,66%
	m.11402T>C	ATC→ACC	p.Ile401Thr	tolerant	61,21%	9,19%	3,14%	26,46%
	m.11572A>C	ATC→CTC	p.Ile458Leu		62,33%	9,64%	3,36%	24,66%

Gene	Sequence variant	Codon change	Amino acid change	SIFT	Alpha helix	Extended strand	Beta turn	Random coil
ND5			**Normal**		60,80%	11,56%	3,35%	24,29%
	m.12063G>A	GTC→ATC	p.Val96Ile	tolerant	59,13%	12,56%	3,35%	24,96%
	m.12330A>G	ACC→GCC	p.Thr185Ala		58,53%	12,88%	3,68%	24,92%
	m.12346T>A	CTA→CAA	p.Leu190Gln		58,53%	12,88%	3,68%	24,92%
	m.12401T>A	AAT→AAA	p.Asn208Lys		59,13%	12,56%	3,35%	24,96%
	m.12636T>C	TTT→CTT	p.Phe287Leu	intolerant	58,53%	12,88%	3,68%	24,92%
	m.12813G>A	GTT→ATT	p.Val346Ile	tolerant	60,13%	12,40%	3,69%	23,79%
	m.13261C>T	ACC→ATC	p.Thr495Ile		60,13%	12,40%	3,69%	23,79%
	m.13299T>A	TCA→ACA	p.Ser508Thr		61,47%	11,89%	3,69%	22,95%
	m.13459T>C	ATC→ACC	p.Ile561Thr		60,97%	11,89%	3,69%	23,45%
ND5/ND6			**Normal**		24,59%	24,59%	14,75%	36,07%
	m.13594G>A	GGG→GAG<br>ACC→ACA	p.Gly606Glu<br>p.Thr172=	tolerant	26,23%	24,59%	11,48%	37,70%
ND6			**Normal**		42,77%	24,28%	8,09%	24,86%
	m.13791T>C	ATT→GTT	p.Ile107Val	tolerant	41,62%	26,01%	8,09%	24,28%
	m.13802G>A	ACT→ATT	p.Thr103Ile		43,35%	23,70%	8,09%	24,86%
CYB			**Normal**		54,45%	8,63%	2,70%	34,23%
	m.14474G>A	GTA→ATA	p.Val98Met	intolerant	53,10%	9,70%	3,23%	33,96%
	m.14534G>A		p.Val118Met		56,72%	7,26%	3,23%	32,80%

*SIFT - sorting intolerant from tolerant ; **The effect of the polymorphism was non-synonymous for ND5 protein, but synonymous for ND6 protein.

Table S8. Detailed information about sequencing data generated using the Illumina MiSeq sequencer.

File name	Format	Num_seqs	Sum_len	Min_len	Avg_len	Max_len	Sequence_ cover
**T015K_R1.fastq.gz**	FASTQ	72,851	18,068,771	35	248,0	301	1,901
**T015K_R2.fastq.gz**		72,851	18,217,335		250,1		1,858
**T015G_R1.fastq.gz**		167,953	39,665,804		236,2		4,175
**T015G_R2.fastq.gz**		167,953	39,960,576		237,9		4,077
**T015TZ_R1.fastq.gz**		116,961	27,365,128		234,0		2,880
**T015TZ_R2.fastq.gz**		116,961	27,585,636		235,9		2,814
**T066K_R1.fastq.gz**		117,081	27,805,504		239,2		2,926
**T066K_R2.fastq.gz**		117,081	28,010,317		241,1		2,858
**T066G_R1.fastq.gz**		71,870	18,012,125		250,6		1,896
**T066G_R2.fastq.gz**		71,870	18,136,049		252,3		1,850
**T066TZ_R1.fastq.gz**		136,624	33,222,812		243,2		3,497
**T066TZ_R2.fastq.gz**		136,624	33,501,479		245,2		3,418
**T087K_R1.fastq.gz**		88,789	22,140,986		250,7		2,330
**T087K_R2.fastq.gz**		88,789	22,260,461		247,5		2,271
**T087G_R1.fastq.gz**		96,235	23,822,852		248,7		2,507
**T087G_R2.fastq.gz**		96,235	23,937,486		228,3		2,442
**T087TZ_R1.fastq.gz**		157,059	35,856,066		228,3		3,774
**T087TZ_R2.fastq.gz**		157,059	36,152,257		230,2		3,689
**T0104K_R1.fastq.gz**		168,095	41,604,329		247,5		4,379
**T0104K_R2.fastq.gz**		168,095	41,899,462		249,3		4,275
**T0104G_R1.fastq.gz**		88,584	21,666,783		244,6		2,280
**T0104G_R2.fastq.gz**		88,584	21,819,602		246,3		2,226
**T0104TZ_R1.fastq.gz**		188,001	42,231,935		224,6		4,445
**T0104TZ_R2.fastq.gz**		188,001	42,530,109		226,2		4,339
**T0105K_R1.fastq.gz**		92,453	21,984,016		237,8		2,314
**T0105K_R2.fastq.gz**		92,453	22,139,645		239,5		2,259
**T0105G_R1.fastq.gz**		128,447	31,821,228		247,7		3,349
**T0105G_R2.fastq.gz**		128,447	32,004,665		249,2		3,265
**T0105TZ_R1.fastq.gz**		66,382	16,902,298		255,9		1,779
**T0105TZ_R2.fastq.gz**		66,382	16,988,422		235,3		1,788
**T0121K_R1.fastq.gz**		101,587	23,907,238		235,3		2,516

File name	Format	Num_seqs	Sum_len	Min_len	Avg_len	Max_len	Sequence_ cover
**T0121K_R2.fastq.gz**	FASTQ	101,587	24,065,145	35	236,9	301	2,455
**T0121G_R1.fastq.gz**		91,531	22,733,289		248,4		2,392
**T0121G_R2.fastq.gz**		91,531	22,896,309		250,1		2,336
**T0121TZ_R1.fastq.gz**		79,573	18,946,244		238,1		1,994
**T0121TZ_R2.fastq.gz**		79,573	19,097,059		240,0		1,948
**T0137K_R1.fastq.gz**		106,514	25,792,408		242,2		2,714
**T0137K_R2.fastq.gz**		106,514	25,925,719		243,4		2,645
**T0137G_R1.fastq.gz**		139,654	33,203,549		237,8		3,495
**T0137G_R2.fastq.gz**		139,654	33,484,318		239,8		3,416
**T0137TZ_R1.fastq.gz**		62,560	15,693,972		250,9		1,651
**T0137TZ_R2.fastq.gz**		62,560	15,829,088		253,0		1,615
**T0138K_R1.fastq.gz**		166,909	40,600,295		243,2		4,273
**T0138K_R2.fastq.gz**		166,909	40,970,517		245,5		4,180
**T0138G_R1.fastq.gz**		124,812	30,090,057		241,1		3,167
**T0138G_R2.fastq.gz**		124,812	30,383,163		243,4		3,100
**T0138TZ_R1.fastq.gz**		114,191	28,382,249		248,6		2,987
**T0138TZ_R2.fastq.gz**		114,191	28,583,244		250,3		2,916
**T0139K_R1.fastq.gz**		642 422	90 200 064		642,4		9,494
**T0139K_R2.fastq.gz**		642 422	90 212 785		642,4		9,205
**T0139G_R1.fastq.gz**		615 566	87 026 295		615, 5		9,160
**T0139G_R2.fastq.gz**		615 566	87 030 463		615, 5		8,880
**T0139TZ_R1.fastq.gz**		111,545	25,702,131		232,7		2,705
**T0139TZ_R2.fastq.gz**		111,545	25,956,174		245,0		2,648
**T0141K_R1.fastq.gz**		128,048	31,721,383		249,7		3,339
**T0141K_R2.fastq.gz**		128,048	31,975,247		245,1		3,262
**T0141G_R1.fastq.gz**		108,327	26,549,659		247,0		2,794
**T0141G_R2.fastq.gz**		108,327	26,757,200		251,5		2,730
**T0141TZ_R1.fastq.gz**		83,358	20,967,542		253,5		2,207
**T0141TZ_R2.fastq.gz**		83,358	21,133,470		237,3		2,156
**T0153K_R1.fastq.gz**		122,476	28,920,728		236,1		3,044
**T0153K_R2.fastq.gz**		122,476	29,124,058		237,8		2,971
**T0153G_R1.fastq.gz**		126,706	30,898,776		243,9		3,252
**T0153G_R2.fastq.gz**		126,706	31,122,536		245,6		3,175

File name	Format	Num_seqs	Sum_len	Min_len	Avg_len	Max_len	Sequence_ cover
**T0153TZ_R1.fastq.gz**	FASTQ	52,392	13,555,452	35	260,3	301	1,426
**T0153TZ_R2.fastq.gz**	FASTQ	52,392	13,639,239	35	240,0	301	1,391
**T0156K_R1.fastq.gz**	FASTQ	128,413	30,819,426	35	240,0	301	3,244
**T0156K_R2.fastq.gz**	FASTQ	128,413	31,142,673	35	242,5	301	3,177
**T0156G_R1.fastq.gz**	FASTQ	81,346	20,099,655	35	247,1	301	2,115
**T0156G_R2.fastq.gz**	FASTQ	81,346	20,238,395	35	248,8	301	2,065
**T0156TZ_R1.fastq.gz**	FASTQ	150,344	35,071,028	35	233,3	301	3,691
**T0156TZ_R2.fastq.gz**	FASTQ	150,344	35,404,302	35	235,5	301	3,612
**T0163K_R1.fastq.gz**	FASTQ	81,931	19,994,696	35	244,0	301	2,104
**T0163K_R2.fastq.gz**	FASTQ	81,931	20,191,809	35	246,4	301	2,060
**T0163G_R1.fastq.gz**	FASTQ	89,750	22,427,998	35	249,9	301	2,360
**T0163G_R2.fastq.gz**	FASTQ	89,750	22,632,832	35	252,2	301	2,309
**T0163TZ_R1.fastq.gz**	FASTQ	23,944	6,273,850	35	262,0	301	660,0
**T0163TZ_R2.fastq.gz**	FASTQ	23,944	6,354,941	35	265,4	301	648,0
**T0195K_R1.fastq.gz**	FASTQ	85,201	21,963,184	35	257,8	301	2,311
**T0195K_R2.fastq.gz**	FASTQ	85,201	22,111,182	35	259,5	301	2,256
**T0195G_R1.fastq.gz**	FASTQ	98,225	24,078,586	35	245,1	301	2,534
**T0195G_R2.fastq.gz**	FASTQ	98,225	24,308,623	35	247,5	301	2,480
**T0195TZ_R1.fastq.gz**	FASTQ	108,187	26,680,851	35	246,6	301	2,808
**T0195TZ_R2.fastq.gz**	FASTQ	108,187	26,879,961	35	248,5	301	2,742
